# Anti-laminin-1 Autoantibodies, Pregnancy Loss and Endometriosis

**DOI:** 10.1080/17402520400001678

**Published:** 2004

**Authors:** Junko Inagaki, Akane Kondo, Luis R. Lopez, Yehuda Shoenfeld, Eiji Matsuura

**Affiliations:** 1 Department of Cell Chemistry Okayama University Graduate School of Medicine and Dentistry Okayama Japan; 2 Department of Obstetric and Gynecology Tokai University Isehara Japan; 3 Corgenix Inc. Westminster CO USA; 4 Department of Medicine B and Center for Autoimmune Diseases Sheba Medical Center hashomer Israel

## Abstract

Laminin-1 is a major component and multifunctional glycoprotein of basement membranes that consists of three different subunits, α1, β1 and γ1 chains. It is the earliest synthesized network-forming protein during embryogenesis and plays an important role in embryonic development, embryonic implantation and placentation. We have recently shown that IgG anti-laminin-1 antibodies were significantly associated with recurrent first-trimester miscarriages and with subsequent pregnancy outcome. Interestingly, these antibodies were also observed in patients with endometriosis-associated infertility but not in patients with other causes of infertility, including tubal factors, hormonal and uterine abnormalities. Laminin-α1, -β1 and -γ1 mRNAs have been detected in 90% of endometriotic lesions and all laminin-α1, -β1 and -γ1 chains were localized in the basement membranes of glandular epithelium in endometriotic peritoneal lesions. Western blot analysis showed that anti-laminin-1 antibodies from those patients reacted with all laminin-1's chains. ELISA also confirmed that one of the target epitopes for these antibodies was located in a particular region of the laminin-1 molecule, i.e. the carboxyl-terminal globular G domain of α1 chain. IgM monoclonal anti-laminin-1 autoantibody, that we recently established, also recognized the G domain. Anti-laminin-1 antibodies from mice immunized with –mouse— laminin-1, caused a higher fetal resorption rate with lower embryonic and placental weights. Thus, anti-laminin-1 antibodies may be important in development of autoimmune-mediated reproductive failures and the assessment of the antibodies may provide a novel non-invasive diagnosis of endometriosis.


*Clinical & Developmental Immunology*, September/December 2004, Vol. 11 (3/4), pp. 261–266

Taylor & Francis healthsciences

### Anti-laminin-1 Autoantibodies, Pregnancy Loss and Endometriosis

JUNKO INAGAKI[a], AKANE KONDO[a,b], LUIS R. LOPEZ[c], YEHUDA SHOENFELD[d] and EIJI MATSUURA[a,*]

[a]*Department of Cell Chemistry, Okayama University Graduate School of Medicine and Dentistry, 2-5-1 Shikata-cho, Okayama 700-8558, Japan;* [b]*Department of Obstetric and Gynecology, Tokai University, Isehara, Japan;* [c]*Corgenix Inc., Westminster, CO, USA;* [d]*Department of Medicine B and Center for Autoimmune Diseases, Sheba Medical Center, Tel-Hashomer, Israel.*

Laminin-1 is a major component and multifunctional glycoprotein of basement membranes that consists of three different subunits, α1, β1 and γ1 chains. It is the earliest synthesized network-forming protein during embryogenesis and plays an important role in embryonic development, embryonic implantation and placentation. We have recently shown that IgG anti-laminin-1 antibodies were significantly associated with recurrent first-trimester miscarriages and with subsequent pregnancy outcome. Interestingly, these antibodies were also observed in patients with endometriosis-associated infertility but not in patients with other causes of infertility, including tubal factors, hormonal and uterine abnormalities. Laminin-α1, -β1 and -γ1 mRNAs have been detected in 90% of endometriotic lesions and all laminin-α1, -β1 and -γ1 chains were localized in the basement membranes of glandular epithelium in endometriotic peritoneal lesions. Western blot analysis showed that anti-laminin-1 antibodies from those patients reacted with all laminin-1's chains. ELISA also confirmed that one of the target epitopes for these antibodies was located in a particular region of the laminin-1 molecule, i.e. the carboxyl-terminal globular G domain of α1 chain. IgM monoclonal anti-laminin-1 autoantibody, that we recently established, also recognized the G domain. Anti-laminin-1 antibodies from mice immunized with "mouse" laminin-1, caused a higher fetal resorption rate with lower embryonic and placental weights. Thus, anti-laminin-1 antibodies may be important in development of autoimmune-mediated reproductive failures and the assessment of the antibodies may provide a novel non-invasive diagnosis of endometriosis.

*Keywords*: Anti-laminin-1 autoantibody; Fetal loss; Recurrent abortion; Infertility; Endometriosis

### INTRODUCTION

Laminin, a multifunctional glycoprotein of basement membranes, consists of three different subunits, α, β and γ chains (Fig. 1) (Burgeson *et al.*, 1994). Laminins are involved in diverse biological activities, including the promotion of cell adhesion, migration, proliferation and differentiation, as well as the formation of the scaffolding network in basement membranes (Colognato and Yurchenco, 2000). These biological processes occur through signal transduction and cell–matrix interactions mediated by laminin-specific receptors and other matrix components. Many of the responsible sites for these activities are localized in the carboxyl-terminal globular G domain of the α chain (Mercurio, 1995; Colognato and Yurchenco, 2000)

To date, at least 15 different isoforms of laminin have been identified and are known to display tissue-specific expression during different stages of embryonic development (Miner *et al.*, 1997; Libby *et al.*, 2000). Laminin-1, composed of α1, β1 and γ1 chains, is the earliest synthesized network-forming component during embryogenesis and plays an important role in embryonic development and placentation. These functions have been confirmed by studies using laminin-α1, -β1 or -γ1 chain or β1-integrin knockout mice (Smyth *et al.*, 1999; Aumailley *et al.*, 2000; Miner *et al.*, 2004).

Laminin-1 from early human embryos increases type IV collagenase expression and is thought to enhance trophoblast adhesion to maternal matrix in the peri-implantation period (Turpeenniemi-Hujanen *et al.*, 1992). In blastocyst or early implanting mouse embryo, laminin-1 is localized in the inner cell mass and in the trophectoderm basement membrane. As implantation proceeds, laminin-1 is expressed in chorionic basement membranes and in Reithert's membrane near the ectoplacental cone (Klaffky *et al.*, 2001; Miner *et al.*, 2004). In human first-trimester placenta, laminin-α1 chain is detected in trophoblastic basement membrane and is in direct contact with extravillous trophoblastic cells. In second-trimester

*Corresponding author. Tel.: +81-86-235-7402. Fax: +81-86-235-7404. E-mail: eijimatu@md.okayama-u.ac.jp

ISSN 1740-2522 print/ISSN 1740-2530 online © 2004 Taylor & Francis Ltd
DOI: 10.1080/17402520400001678

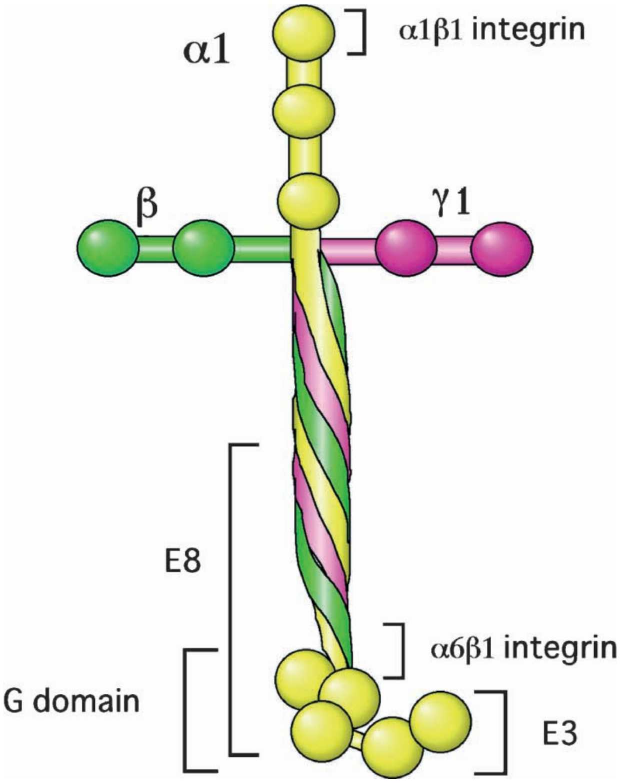

FIGURE 1 Structure of laminin-1. E3 and E8 designate proteolytic (elastase) fragments of laminin-1. Both α1β1 and α6β1 integrins indicate the integrin-binding sites of laminin-α1 chain.

placenta when extravillous trophoblast forms anchoring trophoblastic cell columns, laminin-α1 chain is selectively found at the site where the villous basement membrane is in contact with proliferating cells in trophoblastic islands or columns (Korhonen and Virtanen, 2001). Thus, laminin-1 may have an important role in all processes related to embryogenesis, implantation and placentation.

In the present article, we review the clinical associations of anti-laminin-1 autoantibodies, mainly based on our recent studies.

### AUTOANTIBODIES TO LAMININS IN ANIMALS

Anti-laminin-1 autoantibodies were first detected in the sera of monkeys with history of reproductive failure, and their sera caused abnormalities in cultured rat embryos (Carey and Klein, 1989). It was further demonstrated that immunization of monkeys with mouse laminin-1 or with laminin-1 peptides (i.e. YIGSR or RGD) caused embryotoxicity and infertility/spontaneous abortions (Weeks *et al.*, 1989; Chambers *et al.*, 1995). Passive immunization with rabbit anti-laminin-1 antibodies in pregnant mice induced spontaneous abortions and the antibodies were also localized in both Reichert's membrane and visceral yolk sac endoderm cells of the embryos (Foidart *et al.*, 1983). We also established an animal model that produced high titers of anti-laminin-1 antibodies after immunization with mouse laminin-1. These mice were prone to have a lower successful pregnancy rate. Moreover, these mice had a higher incidence of fetal resorption and decreased placental and embryonic weights (Matalon *et al.*, 2003). Most recently, we have established a mouse IgM monoclonal antibody against the G domain of laminin-α1 from mice immunized with mouse laminin-1 protein as an immunogen (manuscript in preparation), and we are planning to establish an anti-laminin-1 animal model.

### CLINICAL SIGNIFICANCE OF IGG ANTI-LAMININ-1 ANTIBODIES

### Recurrent Abortions

Our recent clinical study showed that IgG anti-laminin-1 antibodies in blood are significantly associated with the recurrent first-trimester miscarriages in humans (Inagaki *et al.*, 2001). All of these observations suggest that IgG anti-laminin-1 antibodies may be responsible for reproductive failure, interfering with an early stage of pregnancy.

A total of 177 recurrent aborters with a history of two or more consecutive first-trimester miscarriages were enrolled into a study and were tested for the presence of anti-laminin-1 antibodies, $\beta_2$-glycoprotein I ($\beta_2$-GPI)-dependent anticardiolipin antibodies, lupus anticoagulants, anti-DNA antibodies and antinuclear antibodies before they conceived again. These recurrent aborters were then followed up during subsequent pregnancies and the outcomes were evaluated relative to their blood test results prior to pregnancy. Fifty-five (31.1%) women out of the 177 recurrent aborters were positive for IgG anti-laminin-1 antibodies. IgG anti-laminin-1 antibody levels (not IgM antibodies) in recurrent aborters were significantly higher than those of healthy pregnant women and healthy non-pregnant women ($P = 0.0043$ and 0.0073, respectively) (Fig. 2). The live birth rate of subsequent pregnancies of IgG anti-laminin-1 autoantibody-positive recurrent aborters was significantly lower than that of IgG anti-laminin-1 autoantibodies-negative recurrent aborters ($P = 0.032$) (Table I). There were no significant relationships observed between IgG anti-laminin-1 antibodies and other autoantibodies tested (Table II). This study demonstrated that IgG anti laminin-1 autoantibodies are closely related to recurrent miscarriages and subsequent pregnancy outcome of recurrent aborters. Our findings suggest that IgG anti-laminin-1 antibodies may have a harmful effect on events at early stages of pregnancy, such as embryonic implantation, embryogenesis, placental vascularization and/or placental nutrient transport.

### Infertility Caused by Endometriosis

Our recent clinical study also showed that IgG anti-laminin-1 antibodies are significantly associated with endometriosis in infertile patients (Inagaki *et al.*, 2003). Sixty-eight infertile patients who underwent

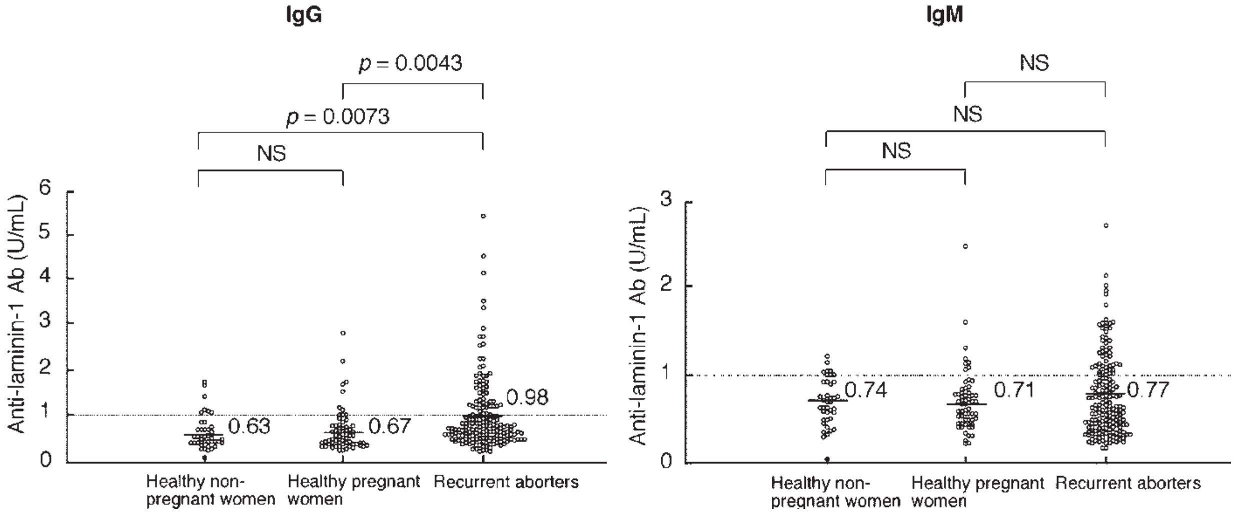

FIGURE 2 Levels of IgG or IgM anti-laminin-1 antibodies in recurrent spontaneous aborters. Anti-laminin-1 antibodies were detected in ELISA using a laminin-1-coated plate. The dotted line shows 1 U/ml (1 U/ml = OD, the mean + 3 SD of healthy non-pregnant women), as a cut off value of the antibodies. Solid lines show the mean value. (Inagaki *et al.*, 2001).

laparoscopy or laparotomy were tested for the presence of IgG anti-laminin-1 antibodies. Twenty infertile patients (29%) were positive for anti-laminin-1 antibodies. Antibody levels in those patients were significantly higher than those in healthy non-pregnant women ($P = 0.0005$) (Fig. 3). The presence of the autoantibodies significantly correlated with endometriosis in those patients ($P = 0.0096$) (Table III). Seventeen of 42 infertile patients with endometriosis (40%) tested positive for anti-laminin-1 antibodies. Other causes of infertility (tubal factor, hormonal and uterine abnormalities and unexplained) were not associated with these antibodies. The values of anti-laminin-1 antibodies were compared between infertile patients with and without endometriosis. Significantly elevated values of the antibodies were observed in 42 infertile patients with endometriosis (the mean value = 1.1 ± 1.2 U/ml) compared to those without endometriosis (0.46 ± 0.33 U/ml) ($P = 0.015$) (Fig. 4).

We demonstrated that infertile patients had significantly higher levels of IgG anti-laminin-1 antibodies. This finding suggested that these antibodies are involved not only in recurrent first-trimester miscarriages but also in infertility in humans. We also showed that these antibodies were strongly associated with infertility, especially when caused by endometriosis. Endometriosis is a widely accepted cause of infertility. A number of studies indicate that infertile patients with endometriosis frequently have the elevated levels of autoantibodies specific for endometrial, ovarian, nuclear antigens and others (Mathur *et al.*, 1982; Pillai *et al.*, 1998; Mathur, 2000). Although, the mechanism(s) of infertility in these disorders is poorly understood, it has been suggested that an aberrant immunological mechanism including the production of autoantibodies might be involved. The presence of anti-laminin-1 antibodies in infertile patients with endometriosis and the function of laminin-1 in embryogenesis, implantation and placentation suggest that anti-laminin-1 antibodies may play a role in modulating very early reproductive processes and be responsible for endometriosis-associated infertility.

From our data, anti-laminin-1 antibodies in infertile patients with endometriosis clearly recognized the G domain of the laminin-$\alpha$1 chain. The G domain contains the recognition sites of several integrin receptors, playing a role in various biological activities (Mercurio, 1995; Colognato and Yurchenco, 2000). It was previously shown that the direct inhibition of laminin-1 binding to integrin receptors and to other basement membrane components by anti-laminin-1 antibodies, impaired the formation of normal basement membranes and epithelial morphogenesis (Kadoya *et al.*, 1995). Therefore, it is possible that these antibodies may also directly interfere

TABLE I Relationship between the prevalence of anti-laminin-1 autoantibodies and subsequent pregnancy outcome in recurrent spontaneous aborters

| | Anti-laminin-1 Ab (IgG) | | | Anti-laminin-1 Ab (IgM) | | |
|---|---|---|---|---|---|---|
| Characteristics | Positive ($N = 38$) | Negative ($N = 85$) | $P$ value | Positive ($N = 40$) | Negative ($N = 83$) | $P$ value |
| Age | 30.0 ± 3.9 | 30.0 ± 3.3 | NS | 29.3 ± 3.0 | 30.8 ± 3.7 | NS |
| Number of previous pregnancy losses | 2.8 ± 1.5 | 2.8 ± 1.3 | NS | 3.0 ± 1.6 | 2.7 ± 1.2 | NS |
| Outcome of subsequent pregnancy | | | | | | |
| Live births | 19 | 59 | | 28 | 50 | |
| Percent live births | 50.0 | 69.4 | 0.032 | 70.0 | 61.0 | NS |

*$P$, Fisher's exact test. (Inagaki *et al.*, 2001).

TABLE II Relationship in the prevalence of anti-laminin-1 autoantibodies, $\beta_2$-GPI-dependent aCL, LA, aDNA and ANA in recurrent aborters

| Anti-laminin-1 Abs (IgG) | aCL | | | | LAC | | | | anti-DNA Abs | | | | ANA | | | |
|---|---|---|---|---|---|---|---|---|---|---|---|---|---|---|---|---|
| | % | Positive | Negative | P* | % | Positive | Negative | P | % | Positive | Negative | P | % | Positive | Negative | P |
| Positive | 31.1 | 1 | 54 | NS | 31.1 | 11 | 44 | NS | 31.1 | 8 | 47 | NS | 31.1 | 12 | 43 | NS |
| Negative | 68.9 | 3 | 119 | | 68.9 | 16 | 106 | | 68.9 | 18 | 104 | | 68.9 | 17 | 105 | |

*P, Fisher's exact test. (Inagaki et al., 2001).

with the function of laminin-1 to disrupt early reproductive stages and be involved in the development of endometriosis. In light of these findings, anti-laminin-1 antibodies might be clinically important in development of autoimmune-mediated reproductive failure and the antibody assessment may provide a novel non-invasive diagnosis of endometriosis.

### LAMININ-1 EXPRESSION

ELISA showed specific autoantibody reactivity to a particular region of the laminin-1 molecule, i.e. $\alpha$1 chain G domain. Laminin-$\alpha$1, -$\beta$1 and -$\gamma$1 mRNAs were also detected in 90% of endometriotic lesions. Immunohistochemical study with specific monoclonal antibodies demonstrated that laminin-$\alpha$1, -$\beta$1 and -$\gamma$1 chains are present in the basement membranes of glandular epithelium in endometriotic peritoneal lesions. Laminin-$\alpha$1 chain was detected only in the basement membranes of glandular epithelium, whereas laminin-$\beta$1 and -$\gamma$1 chains were strongly expressed in the basement membranes of vascular endothelium and in the extracellular matrix of peristromal smooth muscle cells, in addition to the basement membranes of glandular epithelium (manuscript in preparation).

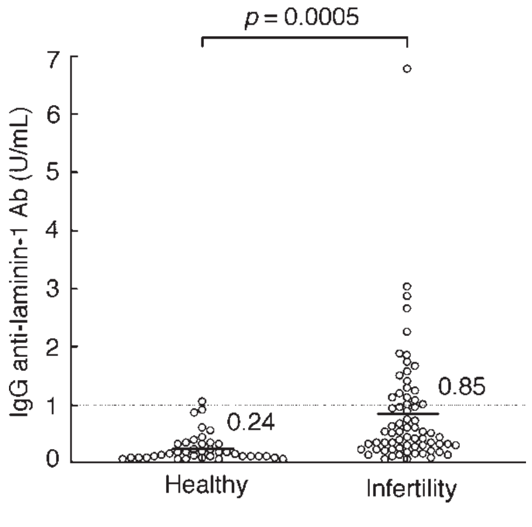

FIGURE 3 IgG values of anti-laminin-1 antibodies in 68 infertile patients who underwent laparoscopy or laparotomy. Anti-laminin-1 antibodies were detected in ELISA using a laminin-1-coated plate. The dotted line shows 1 U/ml (1 U/ml = OD, the mean + 3 SD of healthy non-pregnant women), as a cut off value of the antibodies. Solid lines show the mean value. (Inagaki et al., 2003).

### SPECIFICITY OF ANTI-LAMININ-1 ANTIBODIES

Using Western blot analysis, we showed that anti-laminin-1 antibodies from those patients reacted with all laminin-1's chains, i.e. -$\alpha$1, -$\beta$1 and -$\gamma$1 (Fig. 5). ELISA also confirmed that a target epitope for the antibodies is present in a particular region of the laminin-1 molecule, i.e. carboxyl-terminal globular G domain of $\alpha$1 chain (Fig. 6). It has been

TABLE III Association between IgG anti-laminin-1 autoantibodies and possible causes of infertility in 68 infertile patients who underwent laparoscopy or laparotomy

| Possible cause of infertility | | Anti-laminin-1 Abs Positive (n=20) | Anti-laminin-1 Abs Negative (n=48) | P value |
|---|---|---|---|---|
| Endometriosis | | | | |
| | + | 17 | 25 | 0.0096 |
| | − | 3 | 23 | |
| Tubal factor | | | | |
| | + | 5 | 17 | NS |
| | − | 15 | 31 | |
| Hormonal abnormality | | | | |
| | + | 5 | 11 | NS |
| | − | 15 | 37 | |
| Uterine anomaly | | | | |
| | + | 0 | 2 | NS |
| | − | 20 | 46 | |
| Unexplained | | | | |
| | + | 1 | 10 | NS |
| | − | 19 | 38 | |

P, Fisher's exact test; NS, not significant. (Inagaki et al., 2003).

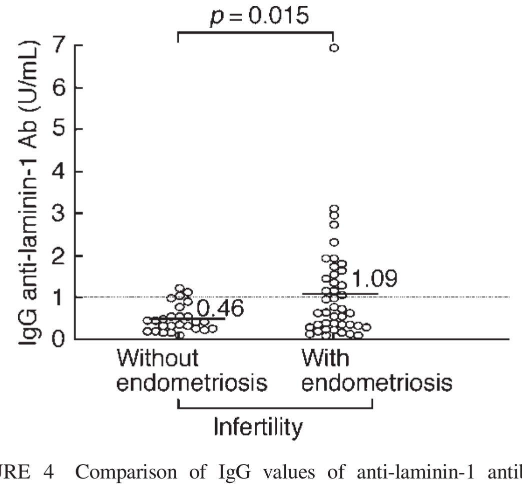

FIGURE 4 Comparison of IgG values of anti-laminin-1 antibodies between infertile patients with and without endometriosis. The dotted and solid lines show the cut off and mean values of the antibodies, respectively. (Inagaki et al., 2003).

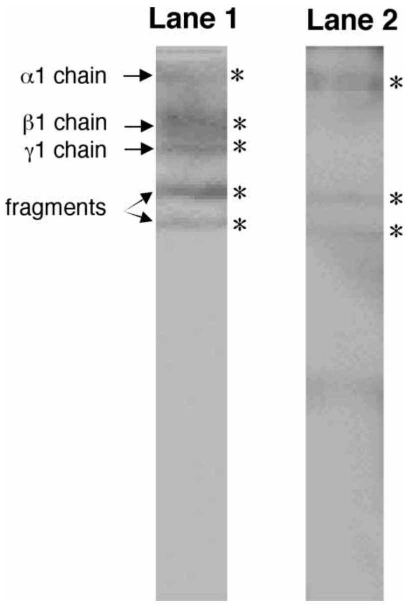

FIGURE 5 Western blot analysis of anti-laminin-1 antibodies. Laminin-1 was run on SDS-PAGE under the reduced condition and transferred on a nitrocellulose membrane. Lane 1: Serum of IgG anti-laminin-1 antibodies positive recurrent aborter. Lane 2: Purified IgM monoclonal antibody (AK-8) to laminin-1.

reported that many responsible sites for various biological activities including promotion of heparin binding, cell attachment and neurite outgrowth are localized in the G domain. Our most interest is whether such anti-laminin-1 antibodies that are associated with fetal loss and/or endometriosis-associated infertility may or may not prevent the cell–cell interaction and impair the formation of normal basement membranes and disrupt early reproductive stages, i.e. whether the antibodies are "pathogenic" or "protective", or just epiphenomenon. As previously described, anti-laminin-1 antibodies induced by active immunization (with mouse laminin-1 as an autoantigen) affected fetal development in the immunized mice. Furthermore, IgM monoclonal autoantibodies to laminin-1 also recognized the G domain. Anyway, laminins seem to be highly immunogenic and antibodies may be polyclonally induced to a variety of epitopic structures on the protein molecules.

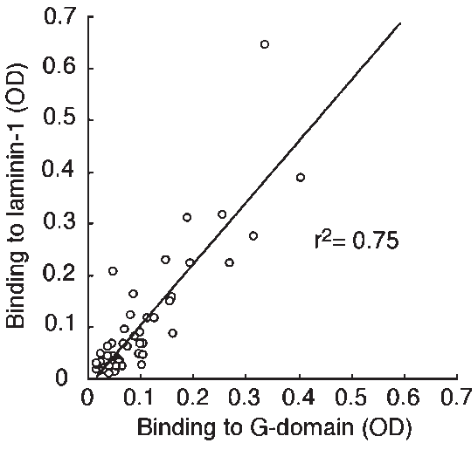

FIGURE 6 Cross-reactivity of IgG anti-laminin-1 autoantibodies from sera of patients with infertility to the intact laminin-1 molecule and to laminin-α1 chain G domain.

### References

Aumailley, M., Pesch, M., Tunggal, L., Gaill, F. and Fassler, R. (2000) "Altered synthesis of laminin 1 and absence of basement membrane component deposition in β1 integrin-deficient embryoid bodies", *J. Cell Sci.* **113**, 259–268.

Burgeson, R.E., Chiquet, M., Deutzmann, R., Ekblom, P., Engel, J., Kleinman, H.K., Martin, G.R., Meneguzzi, G., Paulsson, M. and Sanes, J. (1994) "A new nomenclature for the laminins", *Matrix Biol.* **14**, 209–211.

Carey, S.W. and Klein, N.W. (1989) "Autoantibodies to laminin and other basement membrane proteins in sera from monkeys with histories of reproductive failure identified by cultures of whole rat embryos", *Fertil. Steril.* **51**, 711–718.

Chambers, B.J., Klein, N.W., Conrad, S.H., Ruppenthal, G.C., Sackett, G.P., Weeks, B.S. and Kleinman, H.K. (1995) "Reproduction and sera embryotoxicity after immunization of monkeys with the laminin peptides YIGSR, RGD, and IKVAV", *Proc. Natl Acad. Sci. USA* **92**, 6818–6822.

Colognato, H. and Yurchenco, P.D. (2000) "Form and function: the laminin family of heterotrimers", *Dev. Dyn.* **218**, 213–234.

Foidart, J.M., Yaar, M., Figueroa, A., Wilk, A., Brown, K.S. and Liotta, L.A. (1983) "Abortion in mice induced by intravenous injections of antibodies to type IV collagen or laminin", *Am. J. Pathol.* **10**, 346–357.

Inagaki, J., Matsuura, E., Nomizu, M., Sugiura-Ogasawara, M., Katano, K., Kaihara, K., Kobayashi, K., Yasuda, T. and Aoki, K. (2001) "IgG anti-laminin-1 autoantibody and recurrent miscarriages", *Am. J. Reprod. Immunol.* **45**, 232–238.

Inagaki, J., Sugiura-Ogasawara, M., Nomizu, M., Nakatsuka, M., Ikuta, K., Suzuki, N., Kaihara, K., Kobayashi, K., Yasuda, T., Shoenfeld, Y., Aoki, K. and Matsuura, E. (2003) "An association of IgG anti-laminin-1 autoantibodies with endometriosis in infertile patients", *Hum. Reprod.* **18**, 544–549.

Kadoya, Y., Kadoya, K., Durbeej, M., Holmvall, K., Sorokin, L. and Ekblom, P. (1995) "Antibodies against domain E3 of laminin-1 and integrin α6 subunit perturb branching epithelial morphogenesis of submandibular gland, but by different modes", *J. Cell Biol.* **129**, 521–534.

Klaffky, E., Williams, R., Yao, C.C., Ziober, B., Kramer, R. and Sutherland, A. (2001) "Trophoblast-specific expression and function of the integrin α7 subunit in the peri-implantation mouse embryo", *Dev. Biol.* **239**, 161–175.

Korhonen, M. and Virtanen, I. (2001) "Immunohistochemical localization of laminin and fibronectin isoforms in human placental villi", *J. Histochem. Cytochem.* **49**, 313–322.

Libby, R.T., Champliaud, M.F., Claudepierre, T., Xu, Y., Gibbons, E.P., Koch, M., Burgeson, R.E., Hunter, D.D. and Brunken, W.J. (2000) "Laminin expression in adult and developing retinae: evidence of two novel CNS laminins", *J. Neurosci.* **20**, 6517–6528.

Matalon, S.T., Blank, M., Matsuura, E., Inagaki, J., Nomizu, M., Levi, Y., Koike, T., Shere, Y., Ornoy, A. and Shoenfeld, Y. (2003) "Immunization of naive mice with mouse laminin-1 affected pregnancy outcome in a mouse model", *Am. J. Reprod. Immunol.* **50**, 159–165.

Mathur, S. (2000) "Autoimmunity in endometriosis: relevance to infertility", *Am. J. Reprod. Immunol.* **44**, 89–95.

Mathur, S., Peress, M.R., Williamson, H.O., Youmans, C.D., Maney, S.A., Garvin, A.J., Rust, P.F. and Fudenberg, H.H. (1982) "Autoimmunity to endometrium and ovary in endometriosis", *Clin. Exp. Immunol.* **50**, 259–266.

Mercurio, A.M. (1995) "Laminin receptors: achieving specificity through cooperation", *Trends Cell Biol.* **5**, 419–423.

Miner, J.H., Patton, B.L., Lentz, S.I., Gilbert, D.J., Snider, W.D., Jenkins, N.A., Copeland, N.G. and Sanes, J.R. (1997) "The laminin α chains: expression, developmental transitions, and chromosomal locations of α1-5, identification of heterotrimeric laminins 8–11, and cloning of a novel α3 isoform", *J. Cell Biol.* **137**, 685–701.

Miner, J.H., Li, C., Mudd, J.L., Go, G. and Sutherland, A.E. (2004) "Compositional and structural requirements for laminin and basement membranes during mouse embryo implantation and gastrulation", *Development* **131**, 2247–2256.

Pillai, S., Holt, V., Lee, J.H., Jiang, H. and Rust, P.F. (1998) "Levels of antibodies to transferrin and α2-HS glycoprotein in women with and without endometriosis", *Am. J. Reprod. Immunol.* **40**, 69–73.

Smyth, N., Vatansever, H.S., Murray, P., Meyer, M., Frie, C., Paulsson, M. and Edgar, D. (1999) "Absence of basement membranes after targeting the LAMC1 gene results in embryonic lethality due to failure of endoderm differentiation", *J. Cell Biol.* **144**, 151–160.

Turpeenniemi-Hujanen, T., Ronnberg, L., Kauppila, A. and Puistola, U. (1992) "Laminin in the human embryo implantation: analogy to the invasion by malignant cells", *Fertil. Steril.* **58**, 105–113.

Weeks, B.S., Klein, N.W., Kleinman, H., Fredrickson, T. and Sackett, G.P. (1989) "Laminin immunized monkeys develop sera toxic to cultured rat embryos and fail to reproduce", *Teratology* **40**, 47–57.

